# Enhancing medical students’ self-assessed competence in sexually transmitted infections: outcomes of an interdisciplinary workshop on syphilis, stigmatization and sexual history-taking

**DOI:** 10.1186/s12909-026-10507-0

**Published:** 2026-10-07

**Authors:** Ines Heinen, Nina Matthes, Nina Booken, Martin Härter, Stefan W. Schneider, Finn Abeck

**Affiliations:** 1 Department of Medical Psychology, University Medical Center Hamburg-Eppendorf, Hamburg, Germany; 2 Department of Dermatology and Venereology, University Medical Center Hamburg-Eppendorf, Hamburg, Germany

**Keywords:** Sexually transmitted infections, Dermatology, Dermatology education, Medical students, Undergraduate training, Syphilis

## Abstract

**Background:**

Sexually transmitted infections (STIs), particularly syphilis, continue to pose a significant public health problem. In addition to medical expertise, communication skills – especially when taking a sexual history – are crucial for providing adequate patient care. Previous studies have shown that medical students often feel uncomfortable and inadequately prepared to take a sexual history.

**Methods:**

A pre-post evaluation was conducted as part of a STI workshop for medical students (*n* = 33). The interdisciplinary workshop consisted of dermatological components (epidemiology, clinical presentation, diagnosis, and treatment of syphilis) and psychological components (stigmatization, self-reflection, and sexual history taking), as well as practical exercises (role-playing exercises to practice taking a medical history based on fictional patient cases and simulation of an intramuscular injection using a model). Data was collected using standardized questionnaires immediately before and after the workshop. In addition to the self-assessment scales, an objective test was undertaken concerning the treatment of syphilis and sexual history taking after the workshop.

**Results:**

Thirty-three students participated in the workshop, 31 in the voluntary survey and thus constitute the sample analysed. After the workshop, the students reported a significant increase concerning the self-reported knowledge of epidemiology of syphilis, clinical symptoms, of diagnostic procedures, and of syphilis treatment, as well as in administering an intramuscular injection. Regarding sexual history taking, the students’ answers concerning knowledge of the content, confidence while taking a sexual history, and knowledge of relevance of stigmatization increased significantly. Meanwhile, the self-reported embarrassment while taking a sexual history, and the concerns about patients’ feelings of stigmatization decreased significantly. Most participants stated that that the workshop should be implemented into the standard curriculum.

**Discussion:**

Interdisciplinary teaching formats that combine dermatological and psychological content can help to specifically improve both knowledge and communication skills related to STI care. Teaching these domains together may better prepare medical students for patient-centered sexual healthcare than traditional discipline-specific education.

## Background

Recent epidemiological data demonstrate a global resurgence of sexually transmitted infections (STIs), with syphilis being of particular concern [1, 2]. Syphilis incidence has increased substantially in recent years, reflecting persistent gaps in screening and early diagnosis, as well as prevention and patient education. This phenomenon is particularly concerning considering the wide range of clinical manifestations associated with syphilis, the high transmission rate during unprotected sexual intercourse and the potential of long-term complications if left untreated [3]. In addition, STIs can have an impact on the patient’s psychological, mental and sexual health and can lead to declined Quality of Life, and increased symptoms of anxiety and depression [4].

The increasing burden of STIs highlights the urgent need for healthcare professionals to be adequately trained not only in the diagnosis and treatment of these conditions, but also in effective communication and preventive counselling [5–8].

Despite the growing importance of sexual health in clinical practice, discussing STIs remains associated with stigma and discomfort for both patients and physicians [9]. Physicians often report feeling insecure when taking sexual histories, addressing sexual behavior, and communicating diagnoses related to STIs. Insufficient training in sexual communication and limited exposure to sexual health topics during medical education may contribute to delayed diagnosis of STIs [10].

Previous studies have shown that targeted educational interventions can improve physicians’ confidence and competence in sexual health communication and STI management [5]. However, sexual history-taking and psychosocial aspects of STI care are still underrepresented in medical education. Integrating these topics early during medical training may help to enhance effective sexual history taking and improve future patient care [11, 12].

To address these gaps, we developed an interdisciplinary workshop for medical students combining clinical education on STIs, using syphilis as a model disease, with psychological aspects of sexual health care, including stigmatization, communication strategies, and sexual history-taking. The workshop aimed to strengthen both medical knowledge and interpersonal competencies relevant to STI care. The present study evaluates the impact of this educational intervention on students’ confidence, attitudes and perceived competence regarding sexual health communication and syphilis-related care.

## Methods

### Study design

A pre- and post-questionnaire survey of medical students at Medical University Hamburg (Germany) was conducted in May 2026. The workshop was conducted as part of a diversity symposium for about 140 students in the 1st, 2nd, 3rd and 4th year of the 6-year medical curriculum [13]. The symposium and the subsequent evaluation were coordinated with the Vice Dean’s Office for Teaching. The students could choose one workshop out of eight offered at the same time. The workshops were offered twice on the day of the symposium. A maximum of 17 students could participate in each workshop session. In total, 33 students from all grade levels registered for the two workshops. Prior to study initiation, the study design was reviewed and approved by the local Psychological Ethics Committee of the Centre for Psychosocial Medicine (LPEK-1062). The students were provided an information sheet regarding the study prior to the survey. Survey participation was voluntary and anonymous. The participants did not receive any compensation. Nonparticipation in the survey had no negative consequences for students. Informed consent was obtained from all participants.

The workshop was developed and given by a dermatologist and a psychologist of the University Medical Centre Hamburg-Eppendorf. The workshop was scheduled by the symposium organisation to 140 min each.

### Workshop description

The 140 min workshop was structured into three sections: (1) Epidemiology, clinical presentation, and diagnostics of syphilis (30 min). (2) Stigma and sexual history taking, including practical exercises (60 min). (3) Syphilis treatment combined with hands-on training (30 min), including a 10 min break. The first section covered the epidemiology of syphilis in Germany, transmission pathways, clinical manifestations across disease stages, and diagnostic methods such as serological testing, mainly in lecture-style instruction, including interactive elements using photos of syphilis manifestations within a quiz about STIs. In addition, the workshop highlighted the growing relevance of STIs in clinical practice and discussed behavioral as well as societal factors contributing to rising infection rates. The second section focused on stigma associated with STIs and its effects on patient care, healthcare access, and physician-patient communication. Students reflected on stereotypes, prejudices, and microaggressions in healthcare settings and were introduced to the “5 Ps” framework for sexual history taking (Partners, Practices, Protection from STIs, Past History of STIs, and Pregnancy intention) [14]. Anonymous online feedback was used for content that could be a source of embarrassment in order to gauge students’ attitudes and values. Afterwards, the communication strategies based on 5 Ps were practiced in small-group role-play sessions using fictional patient cases for sexual history taking. Back in the large group, the role-play in small groups was reflected, especially addressing possible feelings of embarrassment and insecurity within the role play as physician and as patient. The final section addressed evidence-based treatment strategies for syphilis, including prevention of Jarisch-Herxheimer reactions. The workshop ended after a practical training for all students in intramuscular injection techniques using models and a feedback session.

### Questionnaire

A paper-based questionnaire was designed by the authors to assess the students’ self-assessed competence regarding STIs and syphilis and attitude towards taking a sexual history interview. Ratings were made on a Likert scale ranging from 1 to 6 (relevance/knowledge “very low” to “very high”), which was based on the regular teaching evaluations of the undergraduate medical curriculum of the University of Hamburg. Thus, the participants were already familiar with the survey method. The students completed the written questionnaire before and after the workshop. The items concerning STIs and sexual history taking underwent peer validation by experienced dermatologists in advance. The items concerning the workshop evaluation were adapted based on the questions from the general teaching evaluation undertaken by the Dean’s Office. The complete questionnaire underwent pilot testing by colleagues. In addition to general student characteristics (age, sex, and current semester of study), prior experience in dermatology, previous training with regard to STIs and sexual history, knowledge about and embarrassment by taking a sexual history as well as knowledge about epidemiological facts, clinical symptoms, diagnoses and therapy of syphilis, and confidence when administering an intramuscular injection were queried at the beginning and end of the workshop. The students answered 5 questions about syphilis and 5 questions about sexual history. At the end of the workshop, its content and structure were evaluated using a 6-point Likert scale (ranging from 1 = “not at all relevant” to 6 = “very relevant”). In addition, students completed a test consisting of three questions designed to objectively assess their knowledge of STI care: (1) highest-risk population for syphilis, (2) the number of penicillin doses required when the time of infection is unknown, and (3) the medication used to prevent Jarisch-Herxheimer reactions. These questions were chosen as the theoretical input to syphilis care focused on these topics and were seen as the fundamental facts for patient care and syphilis treatment. Nevertheless, three questions can’t cover all aspects of syphilis care. Furthermore, students were asked to list the components of the “5 Ps” framework for sexual history taking. For these questions students provided free-text responses.

### Statistics

Data were analysed using GraphPad Prism version 8 (GraphPad Software, San Diego, CA, USA) and IBM SPSS Statistics version 29. Paired sample *t-tests* were used to compare the pre- and post-workshop questionnaires on knowledge and skills. Statistical tests were one-tailed, and the α level of 5% was set at *p* < 0.005 after Bonferroni adjustment for multiple testing. The *t-*tests were one-tailed because participation in the workshop was expected to lead to an increase in self-assessed competence and knowledge, as well as a higher rating of the relevance of the workshop content. A one-tailed test has greater power and the probability of a Type II error (ß error) decreases. The main rationale for the Bonferroni correction is to prevent the accumulation of α errors (inflation of the Type I / α error rate) when conducting multiple statistical tests. If multiple hypothesis tests are performed on the same data, the overall probability (family-wise error rate) of making at least one α error increases with each additional test. This procedure ensures that the overall risk of at least one false-positive result across all tests does not exceed α. The six-point Likert-scale was chosen to copy the format of the regular teaching evaluation, although there is no neutral midpoint and the intervals between the six levels cannot be considered completely equal. To present the intervals as equal as possible, only the outer anchor points were labeled with words, while steps 2 through 5 were represented solely by numbers.

## Results

Of the 33 students, 31 students took part in the voluntary survey (94%). Unless otherwise noted, frequencies and statistical tests refer to *n* = 31 as the final study sample. Of the 31 students, 27 (87.1%) were women, and the mean age was 23.9 ± 3.1 years (range 20–30 years). 10 (32.3%) students were in the 2nd year of medical studies. Only 3 (9.7%) students reported having previous practical experience in dermatology, 10 (32.3%) reported previous theoretical experience in STIs, 12 (38.7%) students reported previous theoretical knowledge about sexual history taking, and 6 (19.4%) students reported that they had already taken a sexual history themselves. Detailed characteristics of the participating students are provided in Table 1.

**Table 1:** General characteristics of the students participating in the workshop on STIs

	Students (*n* = 31)
Age	
Mean value (SD)	23.9 (3.1)
Range	20–30
Sex, *n* (%)	
Female	27 (87.1)
Male	4 (12.9)
Diverse	0 (0.0)
Year and semester, *n* (%)	
1^st^ year, 2^nd^ semester	6 (19.4)
2^nd^ year, 4^th^ semester	10 (32.3)
3^rd^ year, 6^th^ semester	9 (29.0)
4^th^ year, 8^th^ semester	6 (19.4)
Previous training/studies, *n* (%)	
Practical experience in dermatology	3 (9.7%)
Theoretical knowledge about STIs	10 (32.3)
Theoretical knowledge about sexual history taking	12 (38.7)
Practical experience in sexual history taking	6 (19.4)

*SD* standard deviation, *STIs *sexually transmitted infections

**Fig. 1:**
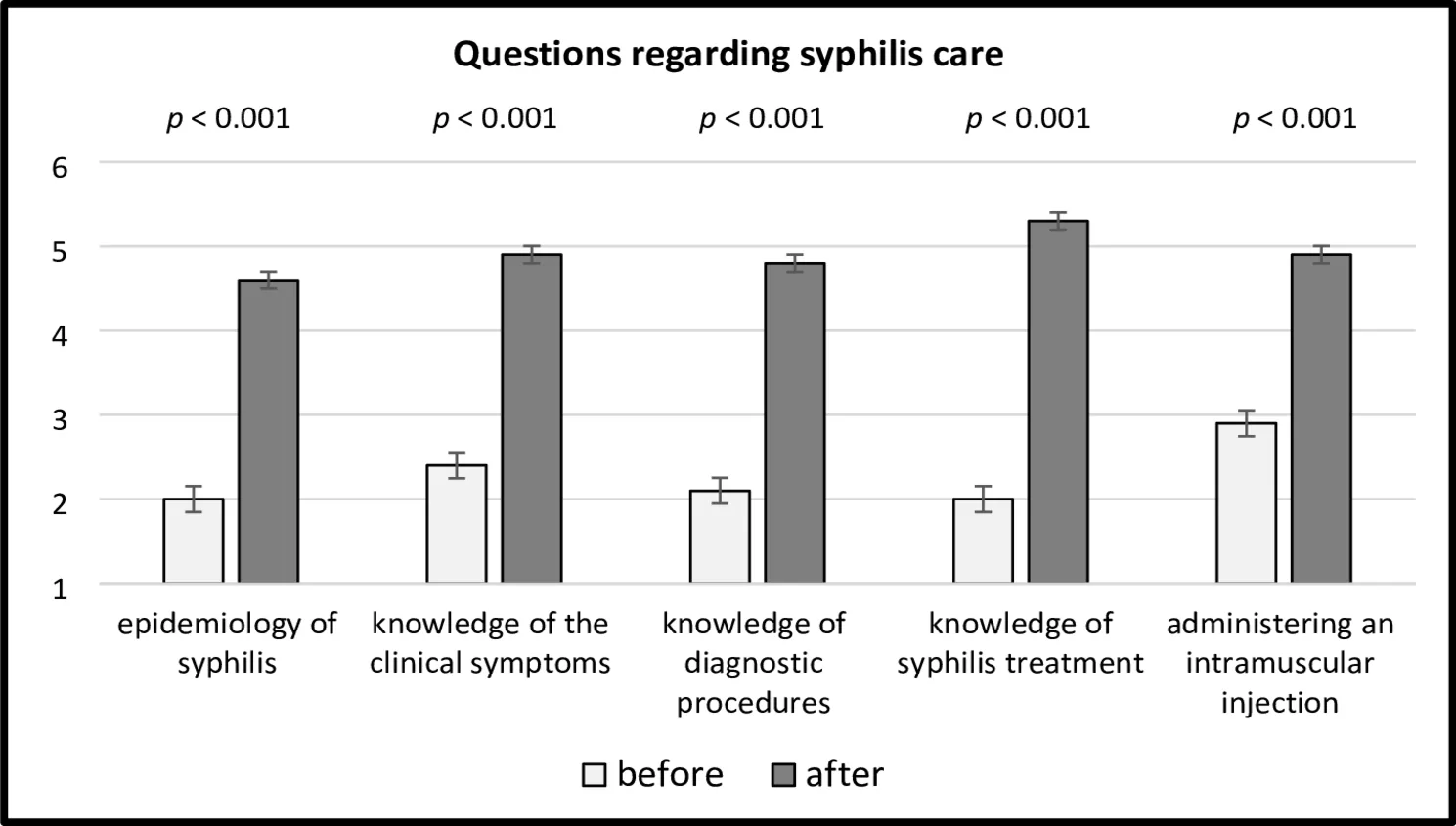
Means and 95% confidence interval of the students’ self-assessment (*n* = 31); survey before and after the workshop concerning knowledge of the epidemiology of syphilis, knowledge of the clinical symptoms of syphilis, knowledge of diagnostic procedures for suspected syphilis, knowledge of syphilis treatment, and confidence when administering an intramuscular injection

Paired-sample *t-tests* were used to compare students’ self-assessment of the 10 items before and after the workshop (Likert scale from 1 “very low” to 6 “very high”), 5 items regarding syphilis care and 5 items regarding sexual history taking. The knowledge of the epidemiology of syphilis was rated on average at 2.0 ± 1.2 before the workshop. The students’ self-assessed knowledge of the clinical symptoms of syphilis averaged 2.4 ± 1.3. After the workshop, the students rated their knowledge of syphilis (4.6 ± 0.8; *t*(30) = -11.661, *p* < 0.001) and of the clinical symptoms (4.9 ± 0.7; *t*(30) = -9.927, *p* < 0.001) as significantly higher than before. Regarding the knowledge of diagnostic procedures for suspected syphilis, a significant increase in knowledge was observed after the workshop according to self-assessment (2.1 ± 1.2 vs. 4.8 ± 0.8; *t*(30) = -12.615, *p* < 0.001). The students’ self-assessed knowledge of syphilis treatment increased significantly from 2.0 ± 1.3 before the workshop to 5.3 ± 0.9 after the workshop (*t*(30) = -11.794, *p* < 0.001). The self-rated confidence when administering an intramuscular injection rose significantly from 2.9 ± 1.7 before the workshop to 4.9 ± 1.0 after the workshop (*t*(30) = -8.211, *p* < 0.001). The results of the questions regarding syphilis care are visualized in Fig. 1.

**Fig. 2:**
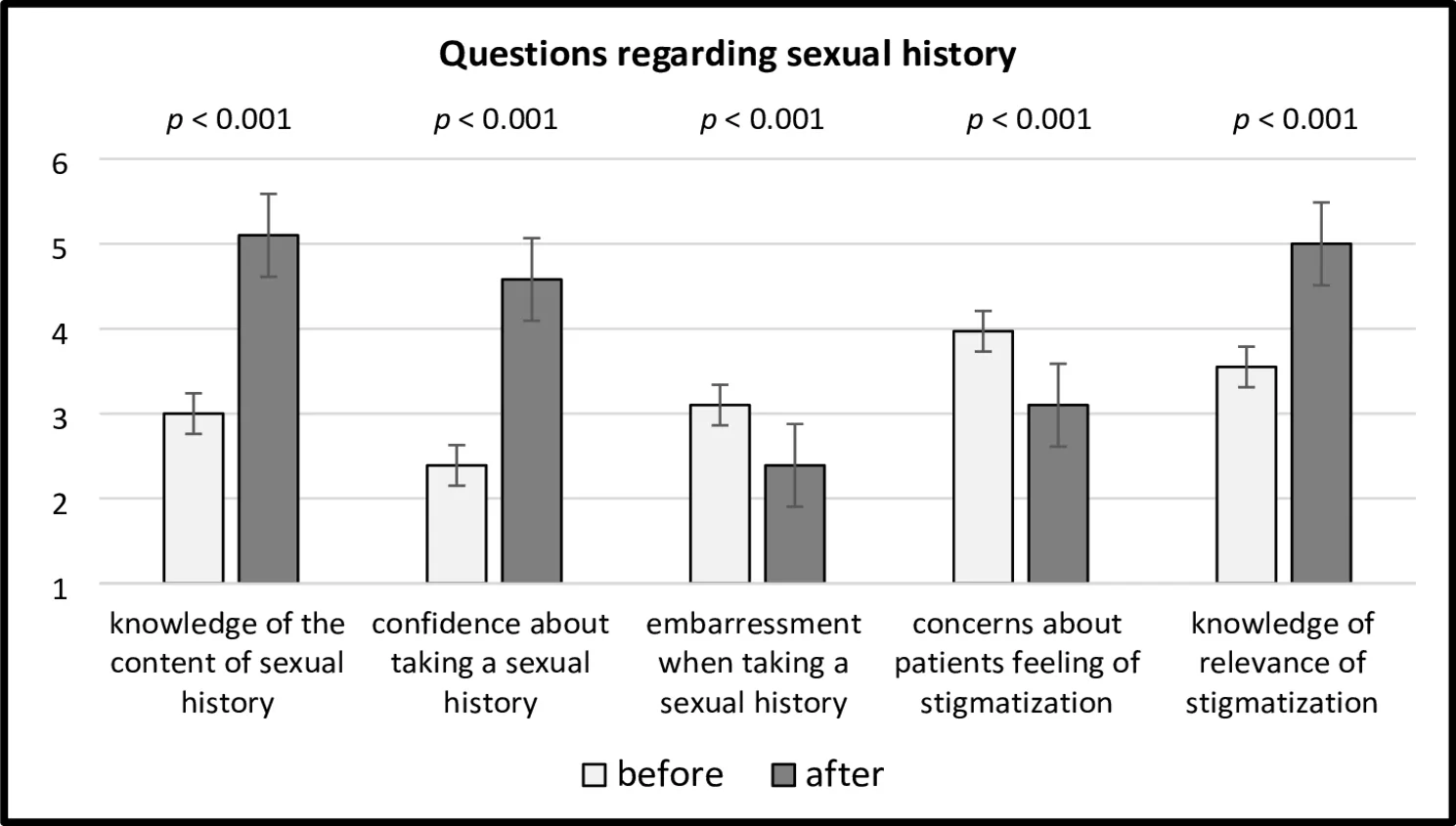
Means and 95% confidence interval of the students’ self-assessment (*n* = 31); survey before and after the workshop, concerning knowledge of the content of a sexual history, students’ self-assessed confidence about taking a sexual history, level of embarrassment when taking a sexual history, students’ concerns about patients’ feelings of stigmatization by taking a sexual history, and self-rated knowledge of relevance of stigmatization among patients with STIs

The knowledge of the content of a sexual history rose significantly from 3.0 ± 1.1 before the workshop to 5.0 ± 0.5 (*t*(30) = -10.231, *p* < 0.001). The students’ self-assessed confidence about taking a sexual history was 2.4 ± 1.1 before and 4.6 ± 0.8 (*t*(30) = -11.021, *p* < 0.001) after the workshop. Regarding the level of embarrassment when taking a sexual history, a significant decrease of embarrassment was observed after the workshop according to self-assessment (3.1 ± 1.2 vs. 2.4 ± 1.3; *t*(30) = 3.803, *p* < 0.001). The students’ concerns about patients’ feelings of stigmatization by taking a sexual history decreased significantly from 4.0 ± 1.1 before the workshop to 3.1 ± 1.0 after the workshop (*t*(30) = 4.338, *p* < 0.001). The self-rated knowledge of relevance of stigmatization among patients with STIs increased significantly from 3.6 ± 1.4 before the workshop to 5.0 ± 0.7 after the workshop (*t*(30) = -5.730, *p* < 0.001). All results of the questions regarding sexual history taking are visualized in Fig. 2. The results of the paired *t*-tests and effect sizes as well as descriptive data are shown in Table 2. The effect sizes of Cohen’s d > 0.8 indicate large effects.

**Table 2:** Descriptive data (mean [M], standard deviation [SD], median [Md], percentile 25 [Q1], and percentile 75 [Q3]) as well as information on the paired-sample t-tests (mean difference [M diff.], 95% confidence interval of the difference [95% CI]) on the self-assessments of the students (n=31) before and after workshop participation regarding syphilis and sexual history, including Cohen's d for effect size

1. Questions regarding syphilis	Before					After					M Diff	95% CI				One-sided p	Cohen's d
	M	SD	Md	Q1	Q3	M	SD	Md	Q1	Q3		Lower value	Upper value				
How would you rate your knowledge of the epidemiology of syphilis?	2.03	1.17	2.00	1.00	3.00	4.61	0.84	5.00	4.00	5.00	-2.58	-3.03		-2.23	<.001		1.23
How would you rate your knowledge of the clinical symptoms of syphilis?	2.35	1.33	2.00	1.00	3.00	4.87	0.67	5.00	4.00	5.00	-2.52	-3.03		-2.00	<.001		1.41
How would you rate your knowledge of the diagnostic procedures for suspected syphilis?	2.06	1.18	2.00	1.00	3.00	4.81	0.75	5.00	4.00	5.00	-2.74	-3.19		-2.30	<.001		1.21
How would you rate your knowledge of syphilis treatment?	2.00	1.34	1.00	1.00	3.00	5.29	0.94	5.00	5.00	6.00	-3.29	-3.86		-2.72	<.001		1.55
How confident do you feel when administering an intramuscular injection?	2.90	1.70	3.00	1.00	4.00	4.94	1.03	5.00	4.00	6.00	-2.03	-2.54		-1.53	<.001		1.38

2. Questions regarding sexual history	Before					After					M Diff	95% CI				One-sided p	Cohen's d
	M	SD	Md	Q1	Q3	M	SD	Md	Q1	Q3		Lower value	Upper value				
How would you rate your knowledge of the content of a sexual history?	3.00	1.13	3.00	2.00	4.00	5.06	0.51	5.00	5.00	5.00	-2.07	-2.47		-1.65	<.001		1.12
How confident do you feel when taking a sexual history?	2.39	1.09	2.00	2.00	3.00	4.58	0.67	5.00	4.00	5.00	-2.19	-2.60		-1.79	<.001		1.11
How would you rate your level of embarrassment when taking a sexual history?	3.10	1.22	3.00	2.00	4.00	2.39	1.26	2.00	2.00	3.00	0.71	0.33		1.09	<.001		1.04
How concerned are you that the patient might feel stigmatized by the sexual history interview?	3.97	1.14	4.00	3.00	5.00	3.10	1.04	3.00	2.00	4.00	0.87	0.46		1.28	<.001		1.12
How would you rate your knowledge of the relevance of stigmatization among patients with STIs?	3.55	1.36	4.00	3.00	5.00	5.00	0.68	5.00	5.00	5.00	-1.45	-1.97		-0.93	<.001		1.41

*STIs* sexually transmitted infections

In the objective knowledge assessment conducted immediately after the workshop, 83.9% of students (*n* = 26) answered all three questions on syphilis care correctly, 48.4% (*n* = 15) correctly listed all five “Ps”, whereas 8 students (25.8%) correctly listed four of the five “Ps”. 6 students ( 19.4%) did not answer this question at all, which was counted as “missing”.

**Fig. 3:**
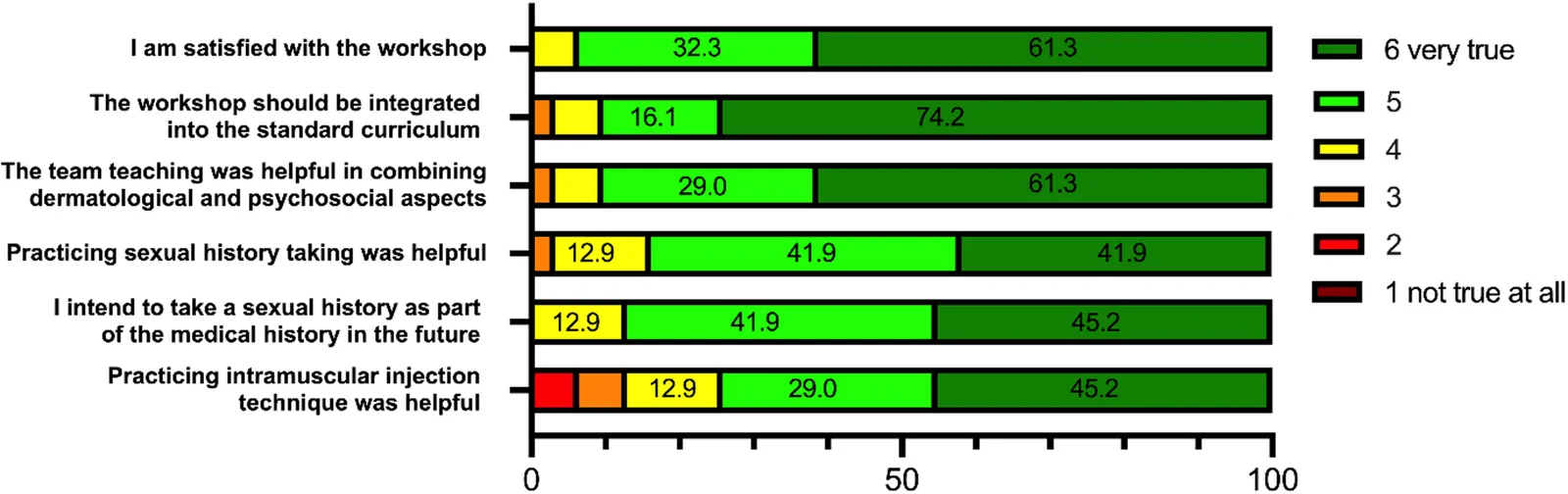
Students’ evaluation of the workshop (*n* = 31; 6-point Likert scale from 1 “not true at all” to 6 “very true”), showing the percentage distribution of responses

The majority of the students were satisfied with the workshop (*n* = 29, 93.6% selected 5 or 6 on a Likert-scale from 1 “not true at all” to 6 “very true”) and stated that the workshop should be implemented into the standard curriculum (*n* = 28, 90.3% with a rating of 5 or 6 on the 6-point Likert scale). Additionally, 87.1% of the respondents indicated that they intend to take a sexual history as part of the medical history in the future (*n* = 27, rating of 5 or 6 on the 6-point Likert scale; Fig. 3).

## Discussion

Due to the global resurgence of sexually transmitted infections, effective interventions are needed to address this growing public health challenge. This study investigated the effects of a newly developed workshop on syphilis, stigmatization, and sexual history taking among undergraduate medical students.

The interdisciplinary workshop significantly improved medical students’ self-assessed knowledge and self-reported confidence in managing syphilis and conducting sexual history taking. Participants reported substantial gains in self-assessed knowledge regarding epidemiology, clinical manifestations, diagnostic procedures, and treatment of syphilis, as well as increased confidence in performing relevant clinical skills such as intramuscular injection. The improvement was not based solely on self-report but was supported by an objective knowledge test, strengthening the findings of this study. Importantly, improvements extended beyond clinical knowledge to psychosocial competencies, with students demonstrating increased self-perceived competence in sexual history taking, a higher awareness of stigma in STI care and less embarrassment when asking patients about sexual health. These findings suggest that integrating clinical dermatological content with structured psychological training may help address emotional and interpersonal barriers to sexual history taking, a well-recognized obstacle to timely STI diagnosis and management [12, 15]. The interdisciplinary approach combining dermatology and psychology may therefore represent a useful approach to STI education, as it addresses both clinical and interpersonal aspects of patient care. These findings are in line with our previous work on stigma in visible skin diseases, suggesting that structured reflection on stigma in visible skin diseases is associated with increased self-reported knowledge of psychological burden and stigma, as well as improved perceived ability to support affected patients [16].

The results of the objective knowledge tests varied widely: while 84% of the students answered the questions on syphilis treatment correctly, only just half (48%) listed all five Ps correctly. This difference may partly reflect the distinct cognitive demands of the two assessment items. The objective questions about syphilis were embedded within a case vignette and provided contextual cues that may have facilitated retrieval of the relevant knowledge, whereas the “5 Ps” question required the unprompted recall of five specific components without contextual support.

To the best of our knowledge, no comparable educational intervention combining dermatological STI teaching with structured psychological training on sexual history taking and stigma awareness has previously been described or evaluated in undergraduate medical education. The high acceptance of the workshop further supports its educational relevance, with more than 90% of participants recommending integration into the undergraduate curriculum. In addition, nearly 90% reported that they intended to routinely incorporate sexual history taking into future clinical practice. According to the 2018 European guideline on the organization of a consultation for STIs, physicians from various specialties (e.g. dermatology, gynecology, urology and family medicine) might be involved in STI care [17]. As sexual history taking, awareness of stigma, and the diagnosis and treatment of STIs are not limited to dermatologists, a thorough knowledge in this field is essential for all future physicians [17, 18]. Accordingly, medical curricula may benefit from incorporating structured, interdisciplinary teaching formats on STIs. Such approaches have the potential to address both clinical knowledge and interpersonal aspects of STI care. We believe that this workshop can be readily transferred to other settings through interdisciplinary collaboration. The 5 Ps concept has been widely published [14]. Lecturers can develop fictional patient cases for role-play or adapt them from the literature. Implementation, including team teaching, requires the involvement of interested lecturers and institutional support for innovative teaching methods.

Several limitations should be acknowledged. The quasi-experimental design without a control group does not allow for causal conclusions. A further limitation is the potential for selection bias. Participation was based on students’ voluntary choice of this workshop among eight workshops offered concurrently at the symposium. Students who selected this workshop may have had a particular pre-existing interest in sexually transmitted infections, dermatology, sexual health, or stigma-related topics and may therefore have been more motivated or receptive to the educational content than students who selected other workshops. In addition, this single-center study conducted at the Medical University of Hamburg limits the generalizability of the findings. Due to the single event of the symposium, only 33 students participated in the workshop, resulting in a relatively small sample size of 31 students who answered the questionnaire. Furthermore, self-assessment within a workshop setting does not necessarily reflect subsequent competence in clinical practice. The objective testing directly after the workshop showed promising results as more than 80% of the students (*n* = 26) answered all three questions on syphilis care correctly, about 50% correctly listed all five “Ps” (*n* = 15) and about 25% students (*n* = 8) correctly listed four of the five “Ps”. However, the long-term retention of knowledge and communication skills remains unknown, as outcomes were assessed immediately after the intervention. In addition, self-assessed competence or knowledge does not necessarily equate to competences shown in real patient contact and are prone to several limitations [19]. For example, the Dunning Kruger effect – overestimating the own abilities and competencies – has been reported frequently in medical education [20]. Future studies should employ controlled, multicenter study designs and include long-term follow-up (for example one year after the intervention), preferably with real or simulated patients, to evaluate the sustained impact of such educational interventions.

## Conclusion

This study demonstrates that an interdisciplinary workshop integrating dermatological and psychological teaching components can significantly enhance medical students’ self-reported knowledge and confidence in the management of STIs, particularly syphilis. Beyond improvements in self-assessed clinical competence, participants reported reduced embarrassment and decreased concerns regarding stigmatization when taking a sexual history, alongside increased awareness of the role of stigma in STI care. These findings support the potential value and acceptability of interdisciplinary, practice-oriented approaches to STI education.

## Data Availability

The data that support the findings of this study are available from the corresponding author upon reasonable request.

## Abbreviations

STI: Sexually transmitted infection
STIs: Sexually transmitted infections
